# Impact of thyroid ultrasonography combined ultrasound-guided fine-needle aspiration biopsy in detection thyroid microcarcinoma: A protocol of systematic review

**DOI:** 10.1097/MD.0000000000021712

**Published:** 2020-08-14

**Authors:** Zhuan-Ning Han, Zhe Liu, Jing Wang

**Affiliations:** a Department of Ultrasound, The Second Affiliated Hospital of Xi’an Medical College, Xi’an; b Department of Cardiology; c Department of Endocrine and Metabolism, Yanan University Affiliated Hospital, Yan’an, China.

**Keywords:** sensitivity, specificity, thyroid microcarcinoma, thyroid ultrasonography, ultrasound-guided fine-needle aspiration biopsy

## Abstract

**Background:**

This study aims to explore the accuracy of thyroid ultrasonography (TUS) combined ultrasound-guided fine-needle aspiration biopsy (UGFNAB) for detection of thyroid microcarcinoma (TMC).

**Methods:**

A comprehensive search in PUBMED, EMBASE, Cochrane Library, Web of Science, Scopus, Chinese Biomedical Literature Database, and China National Knowledge Infrastructure from the beginning to the June 1, 2020 without language and publication status limitations. Two authors will independently perform articles identification, data extraction and assessment of study quality. Any disagreements will be resolved by discussion with a third author. We will carry out study quality evaluation using Quality Assessment of Diagnostic Accuracy Studies tool, and will employ statistical analysis using RevMan V.5.3 and Stata V.12.0 softwares.

**Results:**

We will summarize current evidence to investigate the accuracy of TUS combined UGFNAB in detection of TMC.

**Conclusion:**

The findings of this study may provide helpful evidence of TUS combined UGFNAB in detection of TMC.

**Study registration:**

INPLASY202070048.

## 1 Introduction

Thyroid carcinoma (TC) is the most common endocrine malignant cancer.^[1–4]^ It comprises of papillary carcinoma, follicular carcinoma, Hürthle cell carcinoma, medullary thyroid carcinoma, and anaplastic carcinoma.^[4–6]^ Of those, papillary TC represents the most common type, and accounts for 80% of all cases.^[7,8]^ Papillary thyroid microcarcinoma (TMC) is defined as less than 10 mm in diameter.^[9–11]^ Its treatments vary according its detection and diagnosis.

Thyroid ultrasonography (TUS) and ultrasound-guided fine-needle aspiration biopsy (UGFNAB) are reported to diagnose TMC.^[12–20]^ However, no systematic review has been conducted to explore the accuracy of TUS combined UGFNAB in detection of TMC. Thus, this study will systematically assess the diagnostic accuracy of TUS combined UGFNAB for TMC detection.

## 2 Methods

### 2.1 Objective

This study aims to assess the accuracy of TUS combined UGFNAB in detection of TMC.

### 2.2 Study registration

This study was registered on INPLASY202070048, and it was reported based on the guideline of Preferred Reporting Items for Systematic Reviews and Meta-Analysis (PRISMA) Protocol statement.^[21]^

### 2.3 Eligibility criteria for study selection

#### 2.3.1 Types of studies

We will include all potential case-control studies (CCSs) that examined the accuracy of TUS combined UGFNAB in detection of TMC. There are not limitations related to the basis of language of publications.

#### 2.3.2 Types of participants

Reports of study involved patients with histological-proven TMC will be included in this study, regardless the country, race, age, and gender.

#### 2.3.3 Type of index test

Index test: any forms of TUS combined UGFNAB will be used to detect patients with TMC.

Reference test: participants with histological-proven TMC will be utilized in the control group.

#### 2.3.4 Types of outcome measurements

The primary outcomes include sensitivity and specificity. The secondary outcomes are positive likelihood ratio, negative likelihood ratio, and diagnostic odds ratio.

### 2.4 Data sources and search strategy

#### 2.4.1 Electronic searches

The following resources will be comprehensively searched in PUBMED, EMBASE, Cochrane Library, Web of Science, Scopus, Chinese Biomedical Literature Database, and China National Knowledge Infrastructure from the inception to the June 1, 2020 regardless language and publication status limitations. A draft search strategy for PUBMED is exerted in Table 1. We will modify similar search strategies for other electronic databases.

**Table 1:**
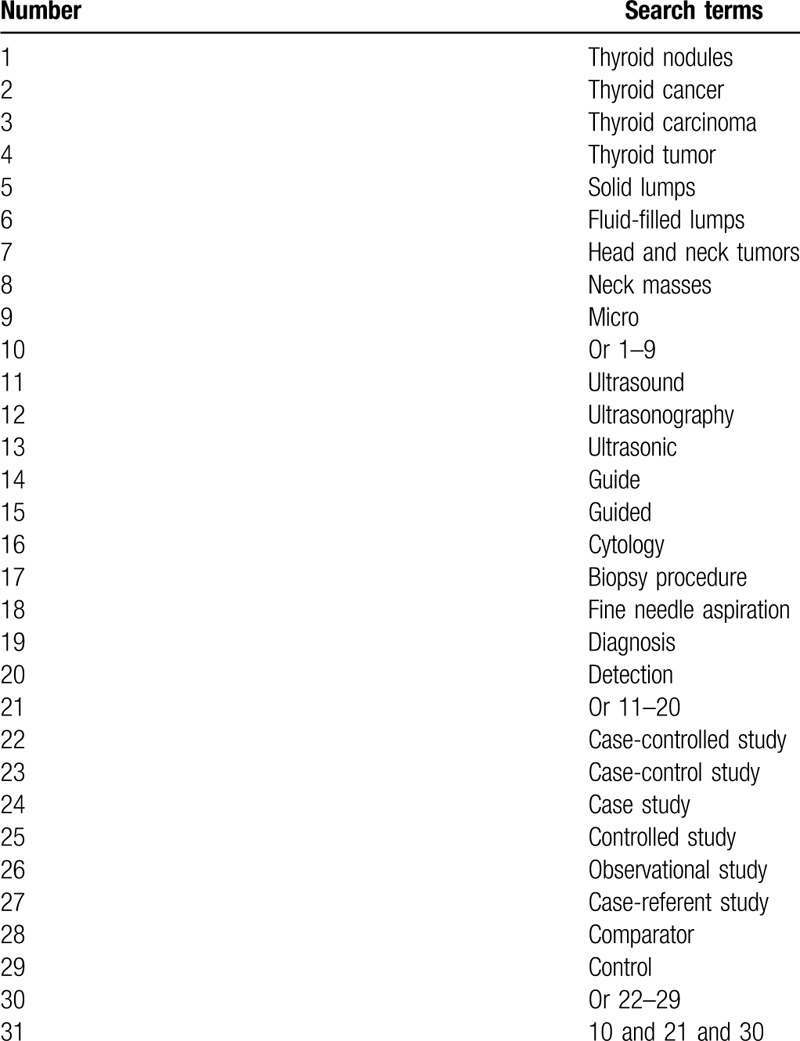
Search strategy of PUBMED.

#### 2.4.2 Other resources

This study will also identify other resources, such as clinical trial registry for ongoing trials, and reference lists of included studies.

### 2.5 Data collection and analysis

#### 2.5.1 Selection of studies

Two authors will screen the titles/abstracts of all identified citations separately, and all duplicates and irrelevant literatures will be removed. Full-text of potential studies will be obtained and read carefully to further examine if they fulfill all eligibility criteria. If disagreements occur between two authors, a third author will be invited to figure out by discussion. The results of study selection will be presented in a PRISMA flow chart.

#### 2.5.2 Data collection and management

Two authors will separately collect data from all included studies by a previous designed data extraction sheet. Any divisions between two authors will be settled by a third author through discussion. The collected information includes study information (e.g., title, first author, time of study, country, study setting, and sample size), study design and methods, patient characteristics (e.g., age, sex, and diagnosis details), details of index and reference tests, outcome indicators, results, and findings.

#### 2.5.3 Dealing with missing data

If any insufficient or missing information is examined, we will contact primary corresponding authors to request it. We will analyze available data if we cannot obtain that data, and will discuss its affects to the study findings.

### 2.6 Methodological quality assessment

Quality Assessment of Diagnostic Accuracy Studies tool^[22]^ will be utilized to appraise the methodological quality for all included studies. Its total score ranges from 0 to 14, with higher score indicating better study quality. Any differences between 2 authors will be solved by discussion with a third author invited.

### 2.7 Statistical analysis

#### 2.7.1 Data synthesis

This study will apply RevMan V.5.3 and Stata V.12.0 softwares to perform statistical analysis. All outcome data will be estimated as descriptive statistics and 95% confidence intervals. We will use *I*^*2*^ statistic test to investigate the heterogeneity across studies. *I*^*2*^ ≤ 50% suggests homogeneity, and a fixed-effects model will be employed. Otherwise, *I*^*2*^ > 50% indicates distinct heterogeneity, and a random-effects model will be placed. We will use collected data to calculate values of sensitivity, specificity, positive likelihood ratio, negative likelihood ratio, and diagnostic odds ratio by 2 × 2 tables. In addition, a descriptive forest plot and a summary receiver operating characteristic plot will be created. Whenever possible, we will carry out a meta-analysis if homogeneity is identified. Otherwise, we will conduct a subgroup analysis to examine the sources of evident heterogeneity. If we cannot perform a meta-analysis after subgroup analysis, we will utilize bivariate random-effects regression approach to plot the estimates of sensitivity and specificity.

#### 2.7.2 Subgroup analysis

If necessary, this study will perform a subgroup analysis to identify possible sources of apparent heterogeneity according to the variations in the study and characteristics, study quality and outcome indicators.

#### 2.7.3 Sensitivity analysis

If possible, this study will carry out a sensitivity analysis to test the robustness of study results by removing low quality studies.

#### 2.7.4 Reporting bias

This study will conduct a funnel plot and Egger regression test to examine reporting bias if over 10 studies are included.^[23]^

### 2.8 Ethics and dissemination

This study will not analyze individual patient data, thus, no ethic approval is necessary. Its results will be published at a peer-reviewed journal.

## 3 Discussion

TMC is a common malignant tumor. Although a variety of diagnosis tool are utilized to detect this disorder, it is still not easy to diagnose at early stage. Previous studies suggested that TUS combined UGFNAB could be used for detection of TMC. However, all results are based on the individual study, and no systematic review specifically investigates the accuracy of TUS combined UGFNAB in detection of TMC. Thus, this systematic review will explore the accuracy of TUS combined UGFNAB in detection of TMC. The results of this study may provide helpful evidence for the clinical diagnosis of TMC.

## Author contributions

**Conceptualization:** Zhuan-Ning Han, Zhe Liu, Jing Wang.

**Data curation:** Jing Wang.

**Formal analysis:** Zhuan-Ning Han, Zhe Liu, Jing Wang.

**Investigation:** Jing Wang.

**Methodology:** Zhe Liu.

**Project administration:** Jing Wang.

**Resources:** Zhuan-Ning Han, Zhe Liu.

**Software:** Zhuan-Ning Han, Zhe Liu.

**Supervision:** Jing Wang.

**Validation:** Zhuan-Ning Han, Jing Wang.

**Visualization:** Zhuan-Ning Han, Jing Wang.

**Writing – original draft:** Zhuan-Ning Han, Zhe Liu, Jing Wang.

**Writing – review & editing:** Zhuan-Ning Han, Zhe Liu, Jing Wang.
